# Antiretroviral drugs from multiple classes induce loss of excitatory synapses between hippocampal neurons in culture

**DOI:** 10.3389/fphar.2024.1369757

**Published:** 2024-03-12

**Authors:** Hannah M. McMullan, Benjamin M. Gansemer, Stanley A. Thayer

**Affiliations:** Department of Pharmacology, University of Minnesota Medical School, Minneapolis, MN, United States

**Keywords:** synapse loss, antiretroviral (ARV), HIV associated neurocognitive disorder, HIV, human immunodeficiency virus, high content imaging (HCI)

## Abstract

**Introduction:** Antiretroviral (ARV) drugs have improved prognoses for people living with HIV. However, HIV-associated neurocognitive disorders (HAND) persist despite undetectable viral loads. Some ARVs have been linked to neuropsychiatric effects that may contribute to HAND. Synapse loss correlates with cognitive decline in HAND and synaptic deficits may contribute to the neuropsychiatric effects of ARV drugs.

**Methods:** Using an automated high content assay, rat hippocampal neurons in culture expressing PSD95-eGFP to label glutamatergic synapses and mCherry to fill neuronal structures were imaged before and after treatment with 25 clinically used ARVs.

**Results and Discussion:** At a concentration of 10 μM the protease inhibitors nelfinavir and saquinavir, the non-nucleoside reverse transcriptase inhibitors etravirine and the 8-OH metabolite of efavirenz, the integrase inhibitor bictegravir, and the capsid inhibitor lenacapavir produced synaptic toxicity. Only lenacapavir produced synapse loss at the nanomolar concentrations estimated free in the plasma, although all 4 ARV drugs induced synapse loss at C_max_. Evaluation of combination therapies did not reveal synergistic synaptic toxicity. Synapse loss developed fully by 24 h and persisted for at least 3 days. Bictegravir-induced synapse loss required activation of voltage-gated Ca^2+^ channels and bictegravir, etravirine, and lenacapavir produced synapse loss by an excitotoxic mechanism. These results indicate that select ARV drugs might contribute to neuropsychiatric effects in combination with drugs that bind serum proteins or in disease states in which synaptic function is altered. The high content imaging assay used here provides an efficient means to evaluate new drugs and drug combinations for potential CNS toxicity.

## Introduction

HIV-1 enters the central nervous system (CNS) early after infection where it establishes a viral reservoir (Kramer-Hammerle et al., 2005; Fois and Brew, 2015; Donoso et al., 2022). HIV infection of the CNS can cause a suite of neurological disorders characterized by cognitive, motor, and behavioral symptoms, collectively referred to as HIV-associated neurocognitive disorders (HAND) (Snider et al., 1983; Antinori et al., 2007). HAND is a spectrum disorder, ranging from asymptomatic impairments that are detectable only in clinical testing to HIV-associated dementia that precludes a patient from living independently (Antinori et al., 2007). Despite the advent of combined antiretroviral therapy (cART) regimens in the mid-1990s and the development of single-tablet regimens in the mid-2000s, approximately half of all people living with HIV (PLWH), youths and adults alike, meet the criteria for a HAND diagnosis at a rate similar to that seen in the pre-cART era (Heaton et al., 2010; Hoare et al., 2016; Saylor et al., 2016; Wang et al., 2020). The persistence of HAND despite suppressive antiretroviral (ARV) therapy is especially troubling because asymptomatic manifestations risk progressing to more severe manifestations over time (Grant et al., 2014) and the population living with HIV is aging.

It is unclear why HAND persists in virologically suppressed patients (Ellis et al., 2023). Cognitive decline in HAND correlates with synapse loss rather than overt neuronal death (Ellis et al., 2007). The presence of active HIV in the CNS has the potential to impair synaptic function through several possible mechanisms, including direct toxicity of viral proteins (Dawson and Dawson, 1994; Kaul and Lipton, 1999; Perez et al., 2001; Nath, 2002; Kim et al., 2008; Shin and Thayer, 2013; Thangaraj et al., 2018), release of inflammatory cytokines (Persidsky et al., 1997; Stoll et al., 2000; Hong and Banks, 2015), and, in the case of patients with a substance use disorder, interactions with concomitant drug use (Silverstein and Kumar, 2014; Hoefer et al., 2015; Mediouni et al., 2015; Sanchez et al., 2015; Fitting et al., 2020; Barbaro et al., 2022). Another potential contributor to HAND persistence is ARV-induced synapse loss. ARVs themselves are associated with a suite of adverse neurological and neuropsychiatric effects, which are correlated with synapse loss (Bennett, 2009; Duman, 2014b; a;Holmes et al., 2019), and these drugs are taken for life. Notably, half of patients taking the non-nucleoside reverse transcriptase inhibitor (NNTRI) efavirenz report experiencing at least one, frequently persistent, neuropsychiatric adverse effect (Molina et al., 2000; Fumaz et al., 2002; Lochet et al., 2003; Fumaz et al., 2005; Arendt et al., 2007). Moreover, integrase strand transfer inhibitor (ISTI), especially dolutegravir, have also been associated with adverse neuropsychiatric effects (Peñafiel et al., 2017; Hoffmann and Llibre, 2019). Previous work has indicated that ARVs can induce dendritic beading, simplification of dendritic processes, and neuronal shrinkage, which are all indicative of neurotoxicity (Robertson et al., 2012). Additionally, it has been shown that efavirenz, an ARV known for its adverse neuropsychiatric side effects, induces dendritic spine loss (Tovar-y-Romo et al., 2012). However, a broad assessment of ARV-induced synapse loss has not been performed. A central challenge to assessing ARV-induced synapse loss is the large number of clinically relevant individual compounds and the multitude of potential cART regimens. To address this challenge, we have utilized a previously described high-content automated image acquisition and analysis system to assess loss of synapses between primary hippocampal neurons in culture (Green et al., 2019). Here, we tested 25 clinically relevant individual ARVs and 6 first-line cART regimens (HIVinfo.NIH, 2023) for their ability to induce the loss of excitatory synapses and, for compounds that tested positive, assessed two potential mechanisms of synaptic toxicity.

## Materials and methods

### Reagents

Materials were obtained from the following sources: Glutamax (catalog number: 35050061), Neurobasal-A medium (catalog number: 12349015), Dulbecco’s modified Eagle’s medium (DMEM; catalog number 31053), Hanks’ balanced salt solution (catalog number: 14175), fetal bovine serum (catalog number: 26140), horse serum (catalog number: 16050), TrypLE™ Express Enzyme (catalog number: 12604039), and penicillin/streptomycin (catalog number: 15140) were from Thermo Fisher Scientific (Carlsbad, CA, United States); N21 Max Media supplement (catalog number: AR008) was from R & D Systems (Minneapolis, MN, United States); glass-bottom 96-well plates (item number: 655892) were from Greiner Bio One (Monroe, NC, United States); bictegravir sodium (BIC, catalog number: ATEH95E04DD4), 2-bromohexadenoic acid (2-BP; catalog number: 238422), 4-aminopyridine (4-AP, catalog number: A-0152), CNQX disodium salt hydrate (catalog number: C239), doravirine (DOR, catalog number: AMBH97F05F7F), MK801 hydrogen maleate (catalog number: M107), nimodipine (Nim, catalog number: N149), cytosine β-D-arabinofuranoside hydrochloide (AraC, catalog number: C6645), Poly-d-lysine (catalog number: P6407), and laminin (catalog number: L2020-1 MG) were from Millipore Sigma (St. Louis, MO, USA); picrotoxin (Pic, catalog number: 1128) was from Tocris-Biotechne (Minneapolis, MN, United States); cabotegravir (CAB, catalog number: 27,215), dolutegravir (DTG, catalog number: 22,191) were from Cayman Chemical (Ann Arbor, MI, United States); 8-OH efavirenz (8-OH EFV, catalog number: U110758) was from Advanced ChemBlocks (Hayward, CA, United States). The following reagents were obtained through the NIH HIV Reagent Program NIAID, NIH: abacavir (ABC, catalog number: ARP-4680), amprenavir (APV, catalog number: ARP-8148), atazanavir sulfate (ATV, catalog number: HRP-10003), darunavir (DRV, catalog number: ARP-11447), efavirenz (EFV, catalog number: HRP-4624), emtricitabine (FTC, catalog number: HRP-10071), enfuvirtide (T-20, catalog number: HRP-12732), etravirine (ETR, catalog number: HRP-11609), lamivudine (3TC, catalog number: ARP-8146), lenacapavir (LEN, catalog number: HRP-20266), lopinavir (LPV, catalog number: HRP-9481), maraviroc (MVC, catalog number: HRP-11580), nelfinavir (NFV, catalog number: ARP-4621), nevirapine (NVP, catalog number: HRP-4666), raltegravir (RAL, catalog number: HRP-11680), rilpivirine (RPV, catalog number: HRP-12417), ritonavir (RTV, catalog number: ARP-4622), saquinavir (SQV, catalog number: ARP-4658), tenofovir (TFV, catalog number: HRP-10199), tipranavir (TPV, catalog number: ARP-11285), zidovudine (AZT, catalog number: ARP-3485).

### Rat hippocampal culture

All animal care and experimental procedures were performed following the Guide for the Care and use of Laboratory Animals published by the U.S. National Institutes of Health. Ethical approval was granted by the Institutional Animal Care and Use Committee of the University of Minneosta (protocol 2209A40405). Hippocampal cultures were prepared from embryonic day 17 Sprague-Dawley rats (Charles River, Wilmington, MA, United States) as described previously (Green et al., 2019) with modifications to plate coating. Each well of a Greiner 96-well plate was coated with 200 µL of 10 μg/mL poly-d-lysine in carbonate buffer (0.1 M sodium carbonate, 0.1 M sodium bicarbonate, tissue-culture grade water, pH 9.6) and left to sit at room temperature for 16–18 h. Plates were then washed three times with carbonate buffer, and each well was coated with 90 µL of 9.667 μg/mL laminin and left to sit at room temperature for at least 4 h. Dissociation and plating of hippocampal neurons were performed as follows. Timed pregnant rats were euthanized by CO_2_ inhalation with an institutionally approved and calibrated CO_2_ chamber. Pups from both sexes were removed and rapidly decapitated with sharp scissors. Hippocampi were removed and placed in cold Ca^2+^ and Mg^2+^-free HEPES-buffered Hanks’ balanced salt solution, followed by trypsinization in 3 mL TrpLE Express for 10 min at 37°C. Trypsin-containing buffer was aspirated and replaced with DMEM without glutamine, supplemented with 10% fetal bovine serum and penicillin/streptomycin (100 U mL^-1^ and 100 mg mL^-1^, respectively), and triturated using flame narrowed Pasteur pipettes to dissociate the cells into a single cell suspension. Immediately prior to plating the neuronal suspension, a glass bottom Greiner 96-well plate, prepared as described above, was washed one time with carbonate buffer followed by a single wash with tissue-grade water. The cell suspension was plated at a density of 35,000 cells/well. On day *in vitro* (DIV) 1, 75% of the media was exchanged with DMEM supplemented with 10% horse serum and penicillin/streptomycin. Cultures were treated with 1 µM AraC on DIV 4 to suppress glial overgrowth. On DIV 8, 75% of media was exchanged with Neurobasal-A medium supplemented with 1% Glutamax and 2% N-21 Max. All cultures were maintained for at least 14 DIV before experiments commenced.

### Virus

Virus mediated gene transfer was used to express postsynaptic density protein 95 fused to enhanced green fluorescent protein (PSD95-eGFP) and mCherry in neurons growing in 96-well plates as previously described (Green et al., 2019). Rat hippocampal cultures were infected with a helper dependent adenovirus (HdAd-hSyn-PSD-95-eGFP-hSyn-mCherry) at a final titer of 1.54 × 10^6^ IGU/mL as part of the DIV 8 75% media change. The cultures used in this study were plated on a laminin-coated substrate at higher density than those described in Green et al. (2019) resulting in an optimal virus titer that is 47% higher.

### Image acquisition

Images were acquired on a Nikon A1 confocal microscope (Nikon, Melville, NY, United States) using a 40 x (0.95 numerical aperture) air objective and controlled with the JOBS module of Nikon Elements software. eGFP was excited at 488 nm and emission detected at 550 nm (50 nm band pass). mCherry was excited at 561 nm and emission detected at 600 nm (50 nm band pass). The confocal aperture was set to 38.5 µm and 1024 × 1024 pixel images scanned at 0.15 µm/pixel. Plates with cells were maintained at 37 °C and 5% CO_2_ in a Chamlide stage-top incubator modified to hold 96-well plates that were mounted on a digitally controlled encoded stage. An acquisition protocol was built in JOBS as described previously (Green et al., 2019). Briefly, the protocol used a plate alignment feature that was run each time a plate was placed on the stage, ensuring repeated imaging of the same image fields. Prior to reading a plate for the first time, an offset from the bottom of the plate to the bottom of the cell layer was determined using an IR-laser-based z-positioning device to find the bottom of the plate; prior to starting each subsequent series the objective was positioned using the previously determined offset to focus on the bottom of the cell layer without interference of any tilt to the plate or warp in its glass bottom. For each region of interest (ROI), red and green image stacks were acquired, each comprised of 10 frames spaced 1 µm apart in the *z*-axis. Multiple ROIs per well were collected. This design allows automatic acquisition of images from the same ROI over time. One well required 6.4 min to image (8 ROIs per well, 10 steps per ROI).

### Image analysis

All images were analyzed in ImageJ (Fiji (imagej.net)) using an updated version of a script originally described in Green et al. (2019). This new version used Groovy scripting and the CLIJ2 package (https://clij.github.io) to take advantage of GPU acceleration and introduced other filtering and thresholding steps to the workflow to improve puncta identification. The script file to be run in Fiji is available at https://github.com/thayerlab. The algorithm generates a binary mask from the mCherry image and then identifies PSD95-eGFP fluorescent puncta within the mask.

The mask generation step was split into two parts. First, a loose threshold was applied to the mask channel using the difference of Gaussian (DoG) blur function (1 minimum, 30 maximum) in CLIJ2, followed by automatic thresholding using the triangle method, to identify any cell soma present in the image. Second, DoG (8 minimum, 9 maximum) and LaPlace filters were followed by thresholding using the triangle method to generate a mask of cell processes that was used to specify the areas in which puncta can be identified. The final mask was generated by combining the soma mask and cell processes mask using a logical NOT function so that the somatic areas were excluded from the mask, and thus further analysis.

To identify synaptic puncta in the eGFP channel, a Gaussian blur function (5) was applied as a low-pass filter to reduce noise in the image. A top hat filter (5 kernel size) was then applied to the blurred image to reduce background and enhance identification of puncta-like structures. After the top hat filter, all pixel values were calculated to the power of 2 to enhance high intensity pixels/regions. Automatic thresholding using the triangle method was then applied to the filtered puncta channel to generate a binary image containing putative synaptic objects. Objects were then filtered by the following criteria: elongation, presence within the cell mask, size, and local signal-noise-ratio (SNR). Elongated objects (i.e., large parts of cell processes with high enough signal to be present after initial filtering and thresholding) were excluded by generating a maximum extension map, in which the maximum distance of any pixel in an object to the centroid of the object was determined. Puncta with elongation values above 10 were excluded. A logical AND function was then applied to the final mask and the remaining puncta to filter out puncta not contained within the cell mask. The remaining puncta were then filtered based on size (6–200 number of pixels/object) prior to SNR calculation and exclusion.

To improve accuracy of puncta identification (and exclusion), particularly in dim images, we employed local SNR exclusion criteria. First, the intensities of puncta objects after elongation, mask, and size exclusion were measured. The local background intensities were measured and a local SNR was calculated for each punctum using the following equation: $SNR=\frac{mean\;punctum\;intensity}{mean\;background\;intensity}$ , where the background intensity is measured from the local background for each punctum, thereby providing local SNRs for each individual punctum. Puncta with SNR values lower than 1.2 were excluded. The remaining puncta were included as the final set of identified puncta.

All intensity measurements were taken from the original puncta channel prior to any processing. Results are presented as the percent change in postsynaptic densities (PSDs) 24, 48, or 72 h after an initial count (t = 0) acquired before drug or control treatments using the following equation:

$\%∆PSDs=\frac{\left[\left(mean\;puncta\;count\;at\;t=24,48\;or\;72\;h\right)\;-\;\left(mean\;puncta\;count\;at\;t=0\;h\right)\right]\left(100\right)}{mean\;puncta\;count\;at\;t=0\;h}$ . Filter settings were optimized to maximize the strictly standardized mean difference in the percent change in puncta counts between untreated and PIC/4-AP–treated ROIs as described previously (Green et al., 2019). All data presented in this study were analyzed with the same script; no adjustments were made for analyzing individual ROIs to avoid adding a subjective element to the analysis.

### Treatment protocols

All experiments used a paired design in which an initial image was collected prior to treatment. Then a 2x concentration of drug or vehicle in cell culture media was applied with a 50% media change. For mechanism studies either 10 µM Nim, an inhibitor of L-type VGCCs, or the combination of 10 µM MK801 and 10 µM CNQX, inhibitors of ionotropic glutamate receptors, were also added at this time. After the treatments were applied the 96-well plate was returned to the cell culture incubator for 18–24 h and then the same ROIs reimaged. For time course experiments the plate was imaged every 24 h for 72 h. The 96-well plate with cultures was maintained at 37°C and 5% CO_2_ in a stage-top incubator during imaging and returned to the cell culture incubator when images were not being acquired. Thus, the long experiments did not affect culture health. Pilot studies suggested that BIC induced synapse loss was not stable after 24 h (12.0% ± 3.4% net difference from untreated at 24 h and 7.6% ± 4.70% difference at 48 h) and thus fresh drug was added at a concentration of 10 µM in a half media change after the 24 and 48 h images were collected. Drug added in the 50% media exchange was at the final concentration so that drug levels never exceeded the initial concentration. Untreated wells also received a half media change.

### Statistics

All statistics were performed using Prism, GraphPad 10 software (La Jolla, CA, United States). An outlier test unique to Prism (robust regression and outlier removal, “ROUT”) was applied to each individual experiment using a Q coefficient of 10%. In pilot studies ROIs identified as outliers typically exhibited large changes in fluorescence intensity, alignment drift or fluorescent debris within the mask. The outlier test provided an efficient and objective mechanism to exclude these ROIs. For time course experiments, each time point underwent its own outlier test, and outliers from one time point were excluded from future time points. Data distributions were first tested using the D’Agostino and Pearson test for normality and Welch’s test for homogeneity of variance. Based on the results of the normality and homogeneity of variance tests, we used the following tests as appropriate: unpaired *t*-test, Welch’s *t*-test, Mann-Whitney *U*-test, one-way analysis of variance (ANOVA) with Sídák’s post-tests, Brown-Forsythe ANOVA with Games-Howell’s post-tests, Kruskal–Wallis test with Dunn’s post-tests, or a two-way ANOVA with Sídák’s post-tests. Each image ROI was defined as an individual sample (n = 1); if data were not normal and variance was considered unequal, data were transformed log(X+101) to restore either normality or a variance considered equal.

### Assay validation

**FIGURE 1:**
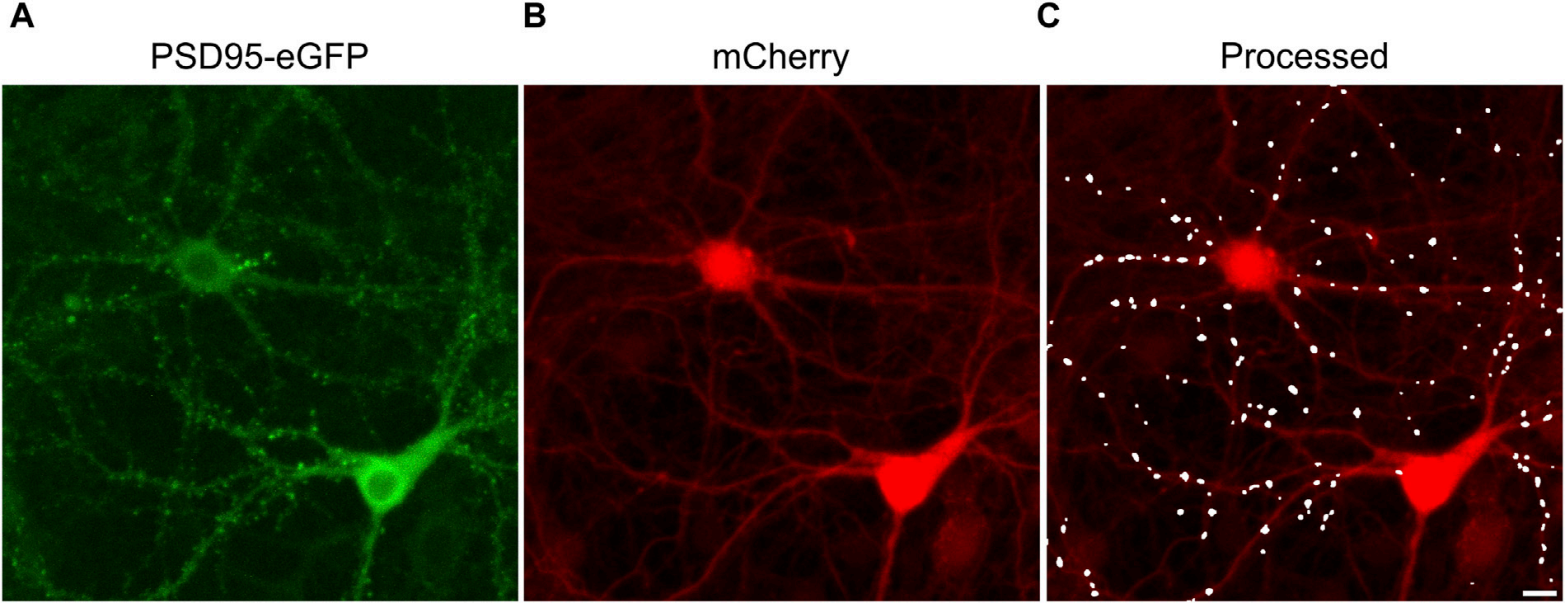
Postsynaptic density protein 95 fused to enhanced green fluorescent protein (PSD95-eGFP) labels postsynaptic terminals at excitatory synapses. **(A,B)** Representative maximum z-projections show images acquired using laser scanning confocal microscopy of rat hippocampal cultures transduced using a bicistronic HdAd virus with a hSyn promoter driving independent expression of PSD95-eGFP **(A)** and mCherry **(B)**. **(C)** Image processing identified fluorescent puncta (PSDs) that were dilated for display purposes and overlaid on the mCherry image (processed). The images are representative of the t = 0 images from the entire study. These specific images were from an ROI on one of the 28 96-well plates used in the screen described in Figure 3. Scale bar indicates 10 µm.

The effects of ARVs on synaptic connections between hippocampal neurons were studied using a previously described automated high content imaging assay (Green et al., 2019). Primary rat neurons in 96-well culture plates were infected with a helper dependent adenovirus (HdAd-hSyn-PSD95-eGFP-hSyn-mCherry) to express PSD95-eGFP to label glutamatergic synapses (Figure 1A) and mCherry to fill neuronal structures (Figure 1B). We have previously shown that 70% of PSDs labeled by the viral product colocalize with the presynaptic matrix protein Bassoon in immunocytochemistry assays, indicating that we are labeling functional synapses (Green et al., 2019). Automated image analysis enabled counting PSD95-eGFP puncta of defined size and intensity in contact with a binary mask derived from the mCherry image (Figure 1C). The identification of puncta by the algorithm uses contrast enhancement, thresholding, and local signal to noise processing that sometimes resolve puncta not easily resolved by eye in a displayed image such as Figure 1A. A detailed description of the image processing can be found in Green et al. (2019). The full frame in Figure 1 displays an individual region of interest (ROI) from which synapses were counted before (t = 0) and after exposure to ARVs or control treatments (t = 24, 48, or 72 h). In subsequent figures only the processed images are displayed.

**FIGURE 2:**
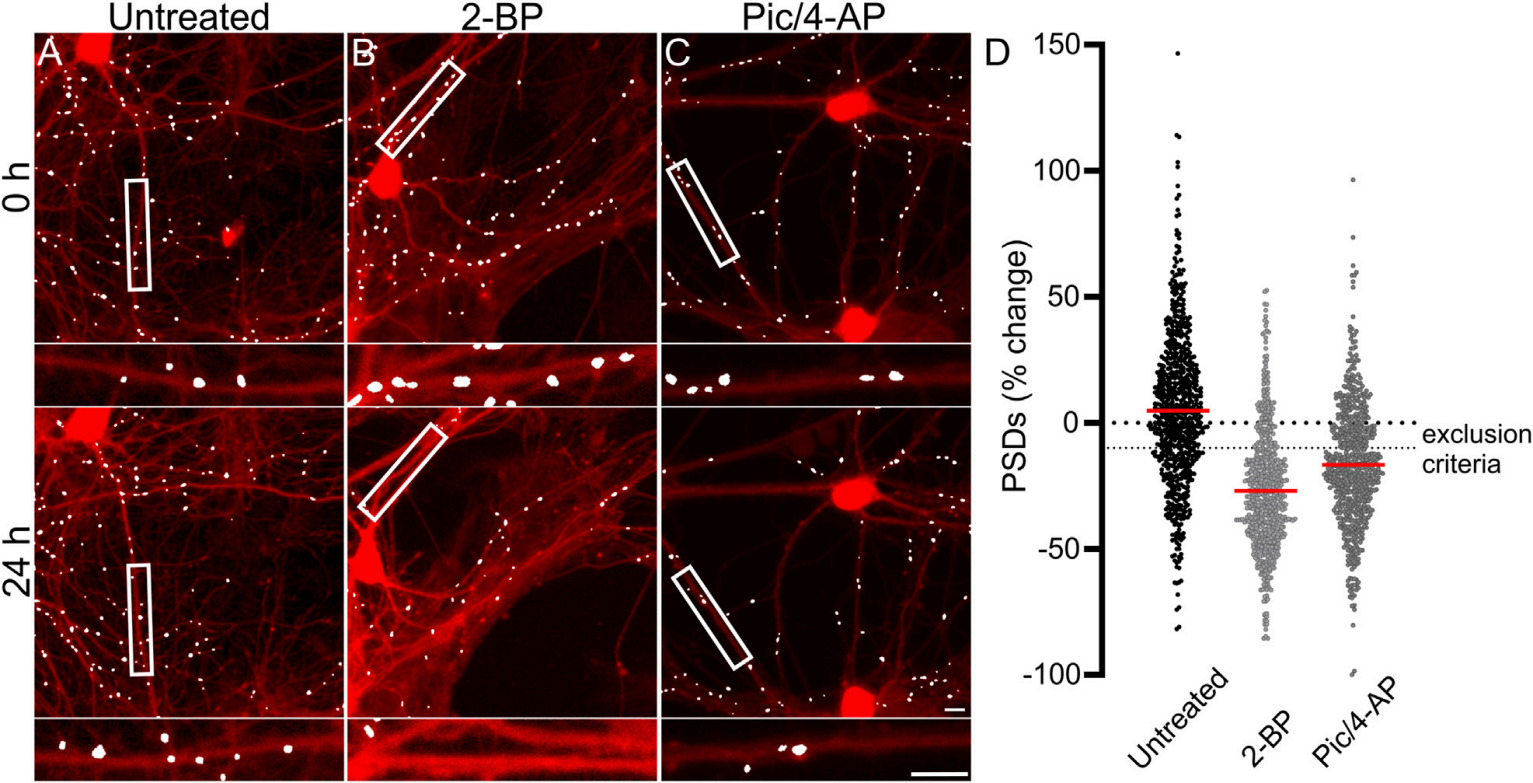
Control experiments validate the synaptic imaging assay. **(A–C)** Representative processed images of ROIs before (t = 0) and after 24 h exposure to no treatment (untreated) **(A)** or treatment with 100 µM 2-BP **(B)**, or 50 µM Pic/2.5 mM 4-AP **(C)**. Insets display enlarged images of the boxed regions. Scale bars indicate 10 µm. **(D)** Dot plots display the change in the number of excitatory synapses (postsynaptic densities (PSDs)) detected as fluorescent PSD95-eGFP puncta for ROIs pooled from the ARV screen shown in Figure 3. Each ROI was imaged as described in Materials and Methods (t = 0) and then a half media change containing no additions (untreated), or the addition of 100 µM 2-BP, or 50 µM Pic/2.5 mM 4-AP (final concentrations are shown). After 24 h the cells were reimaged and the change in count relative to t = 0 plotted. Data are presented as mean ± SEM, with each dot representing one ROI, n = 639–712.

Each experimental run, defined as a single 96-well plate, was validated by testing several control conditions to confirm instrument stability, neuronal culture health and connectivity, and viral gene expression. Changes in synaptic number are presented as a percent change in PSDs for each individual ROI. Because ROIs were objectively chosen by their location within each well of the multi-well plate and not their content, the number of synapses identified within an ROI varied considerably. To avoid amplifying small changes in PSD number by normalizing to a sparse initial puncta count, ROIs with fewer than 50 puncta (t = 0) were excluded from imaging at later time points (t = 24, 48 or 72 h). If more than 50% of the ROIs on a plate expressed less than 50 puncta then the entire plate was excluded. We defined a 10% decrease in the number of identified PSDs in an ROI imaged after 24 h (t = 24 h) relative to an initial PSD count (t = 0 h) to objectively evaluate positive and negative controls (Figure 2). We considered a loss of more than 10% of identified PSDs over 24 h in wells that did not receive any drug treatment (Untreated) to indicate that the culture was compromised and thus the entire plate was excluded from the final data set. To assess the core functionality of our acquisition and analysis assays, after an initial control image was acquired (t = 0) we treated the culture with 2-bromopalmitate (2-BP, 100 µM). This compound inhibits the palmitoylation of PSD95, preventing it from localizing to the cell membrane (Webb et al., 2000; El-Husseini Ael et al., 2002). The assay should not identify diffuse eGFP labelling as PSDs (Figure 2B). If 2-BP failed to induce a loss of at least 10% of identified PSDs over 24 h it was indicative of an instrument, analysis, or viral infection failure, and that plate was excluded. To ensure that the neuronal cultures formed a functional neuronal network they were treated with a combination of the GABA_A_ receptor antagonist picrotoxin (Pic, 50 µM) and the K^+^ channel blocker 4-aminopyridine (4-AP, 2.5 mM) (Figure 2C). Pic/4-AP induces epileptiform activity in synaptically connected networks that results in a downscaling in synaptic number (Müller and Misgeld, 1991; Tatavarty et al., 2013); a failure to induce a loss of at least 10% of identified PSDs over 24 h indicated that the culture failed to develop a functional neuronal network, and that plate was excluded.

**FIGURE 3:**
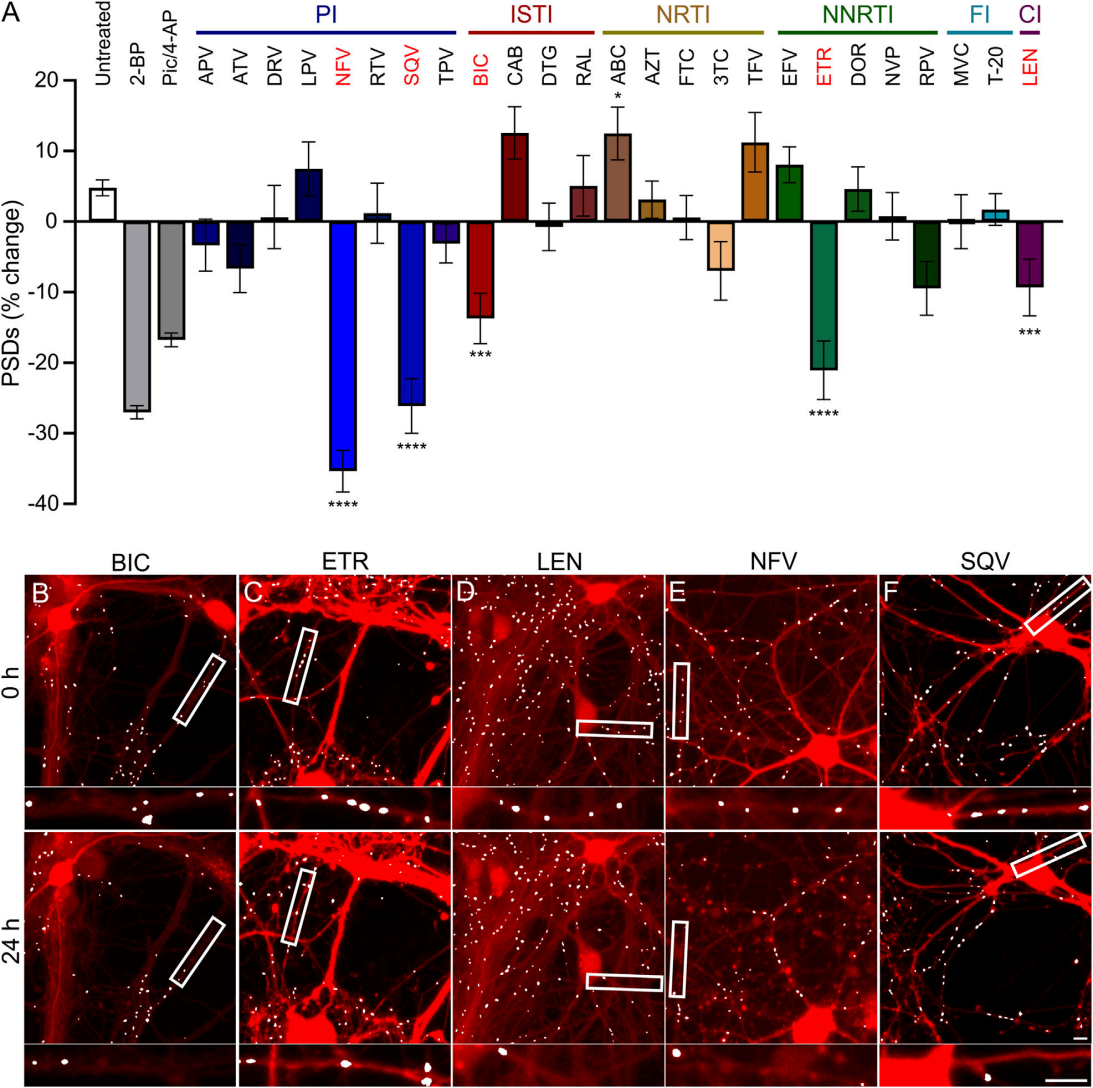
ARVs from four mechanistic classes induce excitatory synapse loss. **(A)** A screen of synapse loss induced by 25 ARVs. After acquisition of an initial image (t = 0) the control treatments (as described in Figure 2) or the indicated ARVs were applied at a concentration of 10 µM. Five antiretrovirals induced statistically significant synapse loss (red): the integrase strand transfer inhibitor (ISTI) bictegravir (BIC), the non-nucleoside reverse transcriptase inhibitor (NNRTI) etravirine (ETR), the capsid inhibitor (CI) lenacapavir (LEN), and the protease inhibitors (PI) nelfinavir (NFV) and saquinavir (SQV). The NRTI abacavir (ABC) significantly increased the number of synapses. Data are presented as mean ± SEM. Each treatment group includes images collected from 12 to 15 wells from 3 separate platings of primary neuronal cultures. *p < 0.05, ***p < 0.001, ****p < 0.0001. ARVs were compared to their matched untreated controls using a standard *t*-Test, Welch’s *t*-Test, or a Mann-Whitney *U*-test as appropriate. BIC: unpaired *t*-Test, n_BIC_ = 80, n_unt_ = 96, t_(174)_ = 3.930, *p* < 0.001. ETR: unpaired *t*-Test, n_ETR_ = 81, n_unt_ = 83, t_(162)_ = 4.117, *p* < 0.0001. LEN: unpaired *t*-Test, n_LEN_ = 64, n_unt_ = 61, t_(123)_ = 5.128, *p* < 0.0001. NFV: unpaired *t*-Test, n_NFV_ = 70, n_unt_ = 80, t_(148)_ = 8.310, *p* < 0.0001. SQV: Welch’s *t*-Test, n_SQV_ = 96, n_unt_ = 85, t_(175.6)_ = 5.153, *p* < 0.0001. ABC: Welch’s *t*-test, n_ABC_ = 83, n_unt_ = 113, t_(146.7)_ = 2.512, *p* < 0.05. Representative processed images for BIC **(B)**, ETR **(C)**, LEN **(D)**, NFV **(E)**, and SQV **(F)** are shown before (t = 0 h) and 24 h after application of drug. Scale bars indicate 10 µm. Abbreviations: 2-bromopalmitate (2-BP), picrotoxin/4-aminopyridine (Pic/4-AP), amprenavir (APV), atazanavir sulfate (ATV), darunavir (DRV), lopinavir (LPV), ritonavir (RTV), tipranavir (TPV), cabotegravir (CAB), dolutegravir (DTG), raltegravir (RAL), zidovudine (AZT), emtricitabine (FTC), lamivudine (3TC), tenofovir (TFV), efavirenz (EFV), doravirine (DOR), nevirapine (NVP), rilipivirine(RPV), maraviroc (MVC), enfuvirtide (T-20).

Figure 2D shows control data pooled from all 96-well plates used in experiments conducted as part of the antiretroviral screen shown in Figure 3. In Figure 2D, the percent change in PSD number for each ROI in the data set is plotted. The number of PSDs in untreated ROIs increased by 5% ± 1% (Figures 2A, D). Increases in the number of PSDs in untreated ROIs are consistent with the continued synaptogenesis that occurs in primary hippocampal cultures as the network matures (Verderio et al., 1999). ROIs treated with 100 µM 2-BP exhibited a loss of 27.0% ± 0.9% of their PSDs (Figures 2B, D). Detecting this loss confirms that the analysis software was able to distinguish synaptic puncta from the diffuse labeling that occurs when PSD95-GFP is not palmitoylated. ROIs treated with 50 µM Pic and 2.5 mM 4-AP lost 17% ± 1% of their PSDs (Figures 2C, D). The synapse loss elicited by Pic + 4-AP requires excitatory synaptic activity and thus requires that a fully formed and functional synaptic network be established, that the PSD95-GFP be correctly expressed, localized and detected, and the network be able to downscale the number of synapses. Thus, these cultures established healthy neural networks and this method of synapse quantification can detect physiological downscaling in response to excess excitatory stimulation (Tatavarty et al., 2013).

## Results

### Antiretroviral drugs from four classes induce excitatory synapse loss *in vitro*

Hippocampal neurons in culture were treated for 24 h with each of the 25 ARV drugs listed in Figure 3. ARVs were added to the culture *via* a half-media change immediately following the acquisition of an initial image (t = 0) to a final concentration of 10 µM. A high drug concentration was chosen for this screen to avoid false negative results; concentration response relationships will be presented for drugs that produce synapse loss in this initial test. Cells were reimaged starting approximately 18–24 h after drug application. Each ARV was tested on at least 3 unique cultures, in 4-5 wells per plate, and 8 ROIs imaged per well. Control data shown in Figure 3 are pooled from all plates used in this screen. However, to identify ARVs that elicited significant synapse loss each drug treatment was compared to untreated wells from its matched cohort of three 96-well plates. Vehicle controls were not different from untreated wells.

Drugs poorly soluble in water were dissolved in DMSO at a final concentration of 0.1% that produced 1.0% ± 5.1% decrease in synapse number which was not different from the 2.9% ± 3.6% decrease observed in matched untreated controls (Student’s t-test: t_(114)_ = 0.30, *p* = 0.77). After screening 25 ARVs, 5 drugs were found to elicit statistically significant loss of postsynaptic densities (PSDs). The protease inhibitors (PI) nelfinavir (NFV) and saquinavir (SQV) reduced the number of synapses by 35% ± 3% and 26% ± 4%, respectively. Representative images in Figures 3E, F show processed images before and after 24 h treatment with NFV and SQV, respectively. Careful inspection of the mCherry fluorescence after 24 h exposure to NFV (Figure 3E) revealed signs of gross toxicity including bleb formation on dendrites and the appearance of necrotic particles in some of the ROIs. The other ARVs that evoked synapse loss did not produce gross toxicity. The density of synaptic connections was not significantly affected by the other PIs tested. While relevant at the initiation of the study, SQV was withdrawn from the market in 2018 and dropped from further study. The integrase strand transfer inhibitor (ISTI) bictegravir (BIC) decreased the number of PSDs by 14% ± 4% (Figures 3A, B). The other ISTIs did not produce synapse loss and interestingly, cabotegravir (CAB) increased the synaptic density, though this effect was not statistically significant. None of the nucleoside/nucleotide reverse transcriptase inhibitors (NRTIs) produced significant synapse loss although lamivudine (3 TC) trended towards loss. NRTIs have known effects on mitochondrial DNA replication, which could be a consideration in 3 TC-induced synapse loss (Werth et al., 1994; Kakuda, 2000), but the small effect size precluded further study with this assay. Interestingly, the NRTI abacavir (ABC) produced a statistically significant increase in the number of PSDs. The non-nucleoside reverse transcriptase inhibitor (NNRTI) etravirine (ETR) decreased the number of PSDs by 21% ± 4% (Figures 3A, C). Rilpivirine (RPV) also trended towards loss but the effect did not reach statistical significance. Based on prior work (Tovar-y-Romo et al., 2012), we expected efavirenz (EFV) to evoke synapse loss, but none was detected. We also evaluated a metabolite of efavirenz shown to potently reduce synaptic spine density; the effects of this metabolite, 8-OH EFV, will be presented in a later section of this report. The fusion inhibitors (FIs) did not affect synaptic density. Maraviroc (MVC) has been shown to improve recovery in some models of neurodegeneration (Servick, 2019) but no positive or negative effects were detected in this assay. The capsid inhibitor (CI) lenacapavir (LEN) decreased the number of synapses by 9% ± 4% (Figures 3A, D).

### Concentration dependent synapse loss evoked by ARVs

**FIGURE 4:**
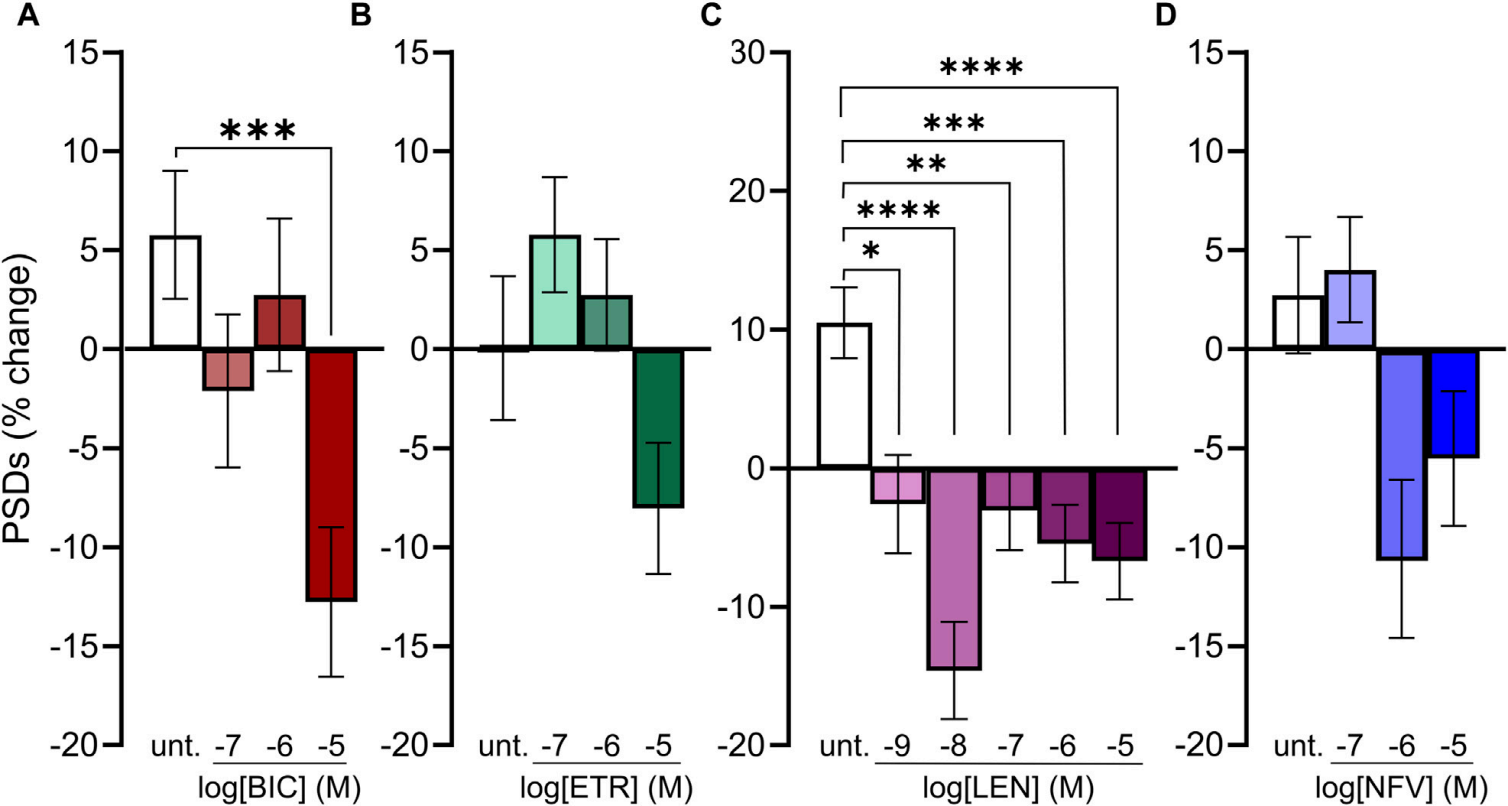
Concentration-response relationships for ARVs that induce synapse loss. **(A–D)** Bar graphs show change in PSDs following 24 h exposure to the treatments indicated. All data are presented as mean ± SEM. *p < 0.05, **p < 0.01, ***p < 0.001, ****p < 0.0001. Each treatment group includes images collected from 12 to 15 wells from 3 separate platings of primary neuronal cultures. **(A)** BIC induced synapse loss at 10 μM. n = 77–88. Kruskal–Wallis test with Dunn’s multiple comparisons tests. Χ^2^_(4)_ = 17.99, *p* < 0.001. **(B)** ETR significantly affected synapse number. n = 88–100. One-way ANOVA. F_(3,378)_ = 3.315, *p* < 0.05. Treatment with 10 µM ETR was not significantly different from Untreated in *post hoc* analysis with Sídák’s multiple comparisons test (*p* = 0.21). **(C)** LEN induced synapse loss at 1 nM. n = 77–145. One-way ANOVA with Sídák’s multiple comparisons test. F_(5,663)_ = 8.117, *p* < 0.0001. **(D)** NFV induced significant synaptic changes (n = 75–86. Kruskal–Wallis test Χ^2^_(3)_ = 9.994, *p* < 0.05) although 1 and 10 µM NFV were not significantly different from untreated in *post hoc* analysis with Dunn’s multiple comparisons test (*p* = 0.055 and *p* = 0.14, respectively).

Because we selected a supratherapeutic ARV concentration (10 µM) for the initial screen, we evaluated decreasing concentrations of ARV drugs that tested positive to determine the lowest concentration that still produced a significant decrease in PSDs in our assay. BIC induced a significant decrease in PSDs only at 10 µM (Figure 4A), which is comparable to total plasma C_max_ but considerably higher than the concentration found free in the plasma or CSF (Table 1) (Gallant et al., 2017; Rigo-Bonnin et al., 2020; Gelé et al., 2021). ETR significantly affected PSD number as indicated by one-way ANOVA of the concentration response data although, the 8.0% ± 3.3% synapse loss induced by 10 µM ETR was not significantly different relative to the 0.1% ± 3.6% change observed in untreated ROIs as determined by Sidak’s posttest (*p* = 0.21) (Figure 4B). ETR induced significant loss relative to matched untreated ROIs in the screen and three other assay series that will be described later in this report. While an ETR-evoked loss of synapses in the 1–10 µM range approximates C_max_, ETR levels free in the serum or CSF are considerably lower (Nguyen et al., 2013; Havens et al., 2020). Of the four ARVs assessed for concentration dependence of PSD loss, only LEN significantly decreased the number of PSDs at concentrations lower than C_max_ (Figure 4C); LEN induced a significant decrease in PSDs at concentrations as low as 1 nM, which is 2 orders of magnitude lower than the reported C_max_ (Table 1) (Link et al., 2020; Paik, 2022). Thus, LEN produces synapse loss at concentrations we estimate as free in the plasma (Table 1). The maximum synapse loss evoked by LEN occurred at a concentration of 10 nM. LEN produced greater synapse loss at 10 nM than either 1 or 100 nM. This unusual concentration response relationship was observed consistently in each of the 3 replicate experiments (plates) and new drug dilutions for each experiment were prepared by serial dilutions in vehicle so that the DMSO concentration was constant at 0.1%. NFV significantly affected PSD number as indicated by Kruskal–Wallis analysis of the concentration response data. Treatment with 1 and 10 µM NFV reduced the PSD count by 10.5% ± 4.0% and 5.5% ± 3.4%, respectively although these decreases were not significantly different relative to untreated ROIs that increased 2.7% ± 2.9% in Dunn’s posttest (*p* = 0.055 and *p* = 0.14, respectively). Review of the puncta counts for ROIs that were excluded by the outlier test found that 4 ROIs that exhibited a complete loss of PSDs were excluded. Visual inspection revealed that there was indeed a complete loss of PSDs in those fields, likely a result of the gross toxicity observed in some ROIs exposed to NFV. To maintain consistency and objectivity across all experiments those ROIs remained excluded.

**TABLE 1:** ARV exposures in clinical studies.

Drug	Class	Dose mg	C_max_ mean ± SD		[ARV]_free plasma_ median (range)		[ARV]_total CSF_ median (range)	
			ng/mL	nM	ng/mL	nM	ng/mL	nM
BIC	ISTI	50 q.d	6080 ± 1325^1^	13,500 ± 2950	10.9 (7.1–20.2)^2^	24.3 (15.8–44.9)	7.14 (4.7–15.0)^2^	15.9 (10.5–33.5)
ETR	NNRTI	200 b.i.d	797 ± 668^3^	1830 ± 1530	6.2 (1.3–47.8)^4^	14.2 (3.0–110)	9.5 (2.0–38.9)^4^	21.8 (4.59–89.4)
LEN	CI	Dosing Regimen 2^5^	124.4 ± 105.9^6^	128.5 ± 109.4	1.87^7^	1.93^7^	n.a	n.a
NFV	PI	1250 b.i.d	4000 ± 800^8^	7050 ± 1410	5.7 (<1.1–17.6)	10(<2.0–31.0)^9^	<1.1 (<1.1–13.1)	<2.0 (<2.0–23.0)^9^

1 Gallant et al., 2017

2 Rigo-Bonnin et al., 2020

3 Havens et al., 2020

4 Nguyen et al., 2013

5 FDA-Sunlenca-Package-Insert, 2022; Days 1 and 2: 600 mg (oral); Day 8: 300 mg (oral); day 15: 927 mg (SC)

6 FDA-Sunlenca-Package Insert, 2022

7 Estimated assuming 1.5% of Cmax is free in plasma

8 FDA-Viracept-Package-Insert, 2005

9 Yilmaz et al., 2006

### Time course of ARV-evoked synapse loss

**FIGURE 5:**
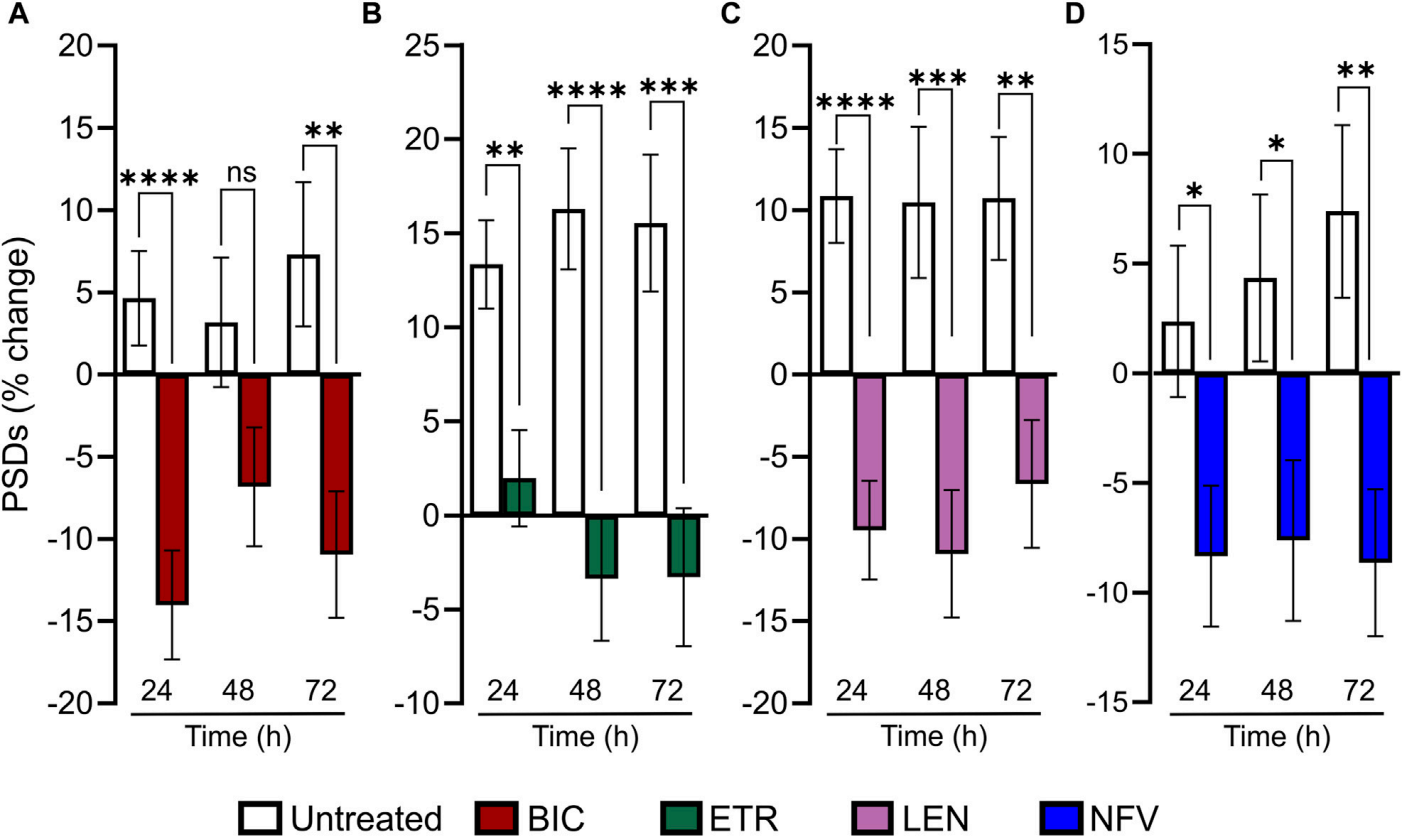
ARV induced synapse loss occurs within 24 h and persists to 72 h **(A–D)** Bar graphs show change in PSDs following 24, 48, and 72 h exposure to the treatments indicated. All PSD changes (t = 24, 48 and 72 h) are presented as a percentage of PSDs counted before treatment (t = 0). All data are presented as mean ± SEM. *p < 0.05, **p < 0.01, ***p < 0.001, ****p < 0.0001. Each treatment group includes images collected from 12 to 15 wells from 3 separate platings of primary neuronal cultures. **(A)** 10 µM BIC induced a decrease in PSDs at 24 h and 72 h. Student’s *t*-test 24 h: n_unt_ = 125, n_BIC_ = 82 t_(205)_ = 4.198, *p* < 0.0001. 48 h: n_unt_ = 95, n_BIC_ = 82 t_(175)_ = 1.849, *p* = 0.066. 72 h: n_unt_ = 94, n_BIC_ = 82 t_(174)_ = 3.080, *p* < 0.01. **(B)** 10 µM ETR induced a persistent decrease in PSDs to 72. 24 h: Student’s *t*-test n_unt_ = 75, n_ETR_ = 78 t_(151)_ = 3.267, *p* < 0.01. 48 h: Mann-Whitney n_unt_ = 74 n_ETR_ = 74 U = 1655, *p* < 0.0001. 72 h: Student’s *t*-test n_unt_ = 74 n_ETR_ = 70, t_(142)_ = 3.683 *p* < 0.001. **(C)** 0.01 µM LEN induced a persistent decrease in PSDs to 72 h. Student’s t-test 24 h: n_unt_ = 102, n_LEN_ = 94, t_(194)_ = 4.908, *p* < 0.0001. 48 h: n_unt_ = 75, n_LEN_ = 69, t_(142)_ = 3.520, *p* < 0.001. 72 h: n_unt_ = 75, n_LEN_ = 68, t_(141)_ = 3.219, *p* < 0.01. **(D)** 10 µM NFV induced a persistent decrease in in PSDs to 72 h. 24 h: Student’s *t*-test n_unt_ = 90, n_NFV_ = 104, t_(192)_ = 2.268, *p* < 0.5. 48 h: Student’s *t*-test n_unt_ = 90, n_NFV_ = 102, t_(190)_ = 2.264 *p* < 0.05. 72 h: Mann-Whitney *U* test n_unt_ = 88, n_NFV_ = 100, U = 3252, *p* < 0.01.

Much as HIV infection is a lifelong condition, so too is ARV therapy a chronic treatment regimen. To assess the effect of ARVs on PSDs over time, we assessed changes in PSDs for 72 h (Figure 5). All PSD changes (t = 24, 48 and 72 h) are presented as a percentage of PSDs counted before treatment (t = 0). The BIC-induced decrease in PSDs developed by 24 h and persisted to 72 h (Figure 5A). ETR induced a statistically significant decrease in PSDs relative to an increase in synapses in untreated wells that persisted to 72 h (Figure 5B). LEN (0.01 µM) induced a statistically significant decrease in PSDs that persisted to 72 h (Figure 5C). The LEN-induced synapse loss remained stable over the first 48 h following administration and persisted for 72 h post administration. Like both ETR and LEN, NFV induced a statistically significant decrease in PSDs at 24 h, and the effect persisted to 72 h (Figure 5D). While the loss from 0 h remained stable in wells treated with NFV, the drug-free wells continued to produce more PSDs, increasing the relative effect of NFV over 72 h. Overall, we did not observe a strong time dependent change in synapses after the initial 24 h treatment with ARV drugs, suggesting that ARV-induced changes in the number of synapses equilibrate to a new set point.

### The role of L-type Ca^2+^ channels in ARV evoked synapse loss

**FIGURE 6:**
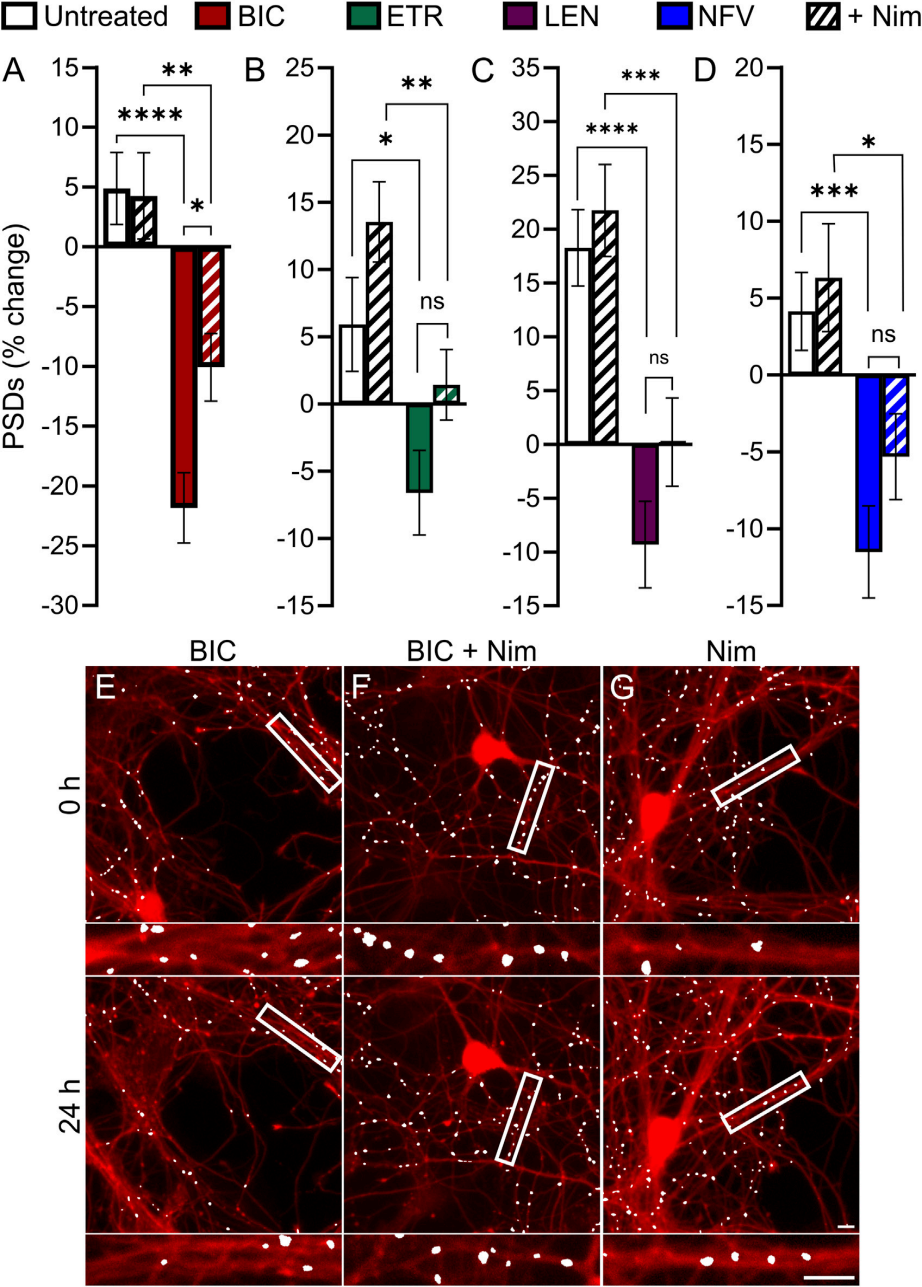
BIC induced synapse loss through L-type VGCCs. **(A–D)** Bar graphs show change in PSDs following 24 h exposure to the treatments indicated. All data are presented as mean ± SEM. *p < 0.05, **p < 0.01, ***p < 0.001, ****p < 0.0001. Each treatment group includes images collected from 12 to 15 wells from 3 separate platings of primary neuronal cultures. **(A)** BIC induced synapse loss was attenuated significantly by nimodipine. n = 73–80. Two-way ANOVA with Sídák’s multiple comparisons test. BIC treatment F_(1,303)_ = 43.87, *p* < 0.0001, Nim treatment F_(1,303)_ = 3.225, *p* = 0.073, interaction effect F_(1,303)_ = 3.986, *p* < 0.05. **(B)** ETR induced synapse loss was not significantly different in the presence of Nim. n = 80–103 Kruskal–Wallis test with Dunn’s multiple comparisons test. Χ^2^_(4)_ = 20.71, *p* < 0.0001. **(C)** LEN induced synapse loss was not significantly different in the presence of Nim. n = 61–71. Two-way ANOVA with Sídák’s multiple comparisons test. LEN treatment F_(1,256)_ = 37.25, *p* < 0.0001, Nim treatment F_(1,256)_ = 2.619, *p* = 0.1068, interaction effect F_(1,256)_ = 0.5642, *p* = 0.4533. **(D)** NFV induced synapse loss was not significantly different in the presence of Nim. n = 63–80. Two-way ANOVA with Sídák’s multiple comparisons test. NFV treatment F_(1,293)_ = 20.71, *p* < 0.0001, Nim treatment F_(1,293)_ = 1.955, *p* = 0.1631, interaction effect F_(1,293)_ = 0.4440, *p* = 0.5057. Representative processed images show ROIs before (t = 0) and after 24 h treatment with BIC **(E)**, BIC + Nim **(F)**, and Nim **(G)**. Insets display enlarged images of the boxed regions. Scale bars indicate 10 µm.

Previous studies have demonstrated a role for L-type voltage gated calcium channels (VGCC) in the loss of dendritic spines and axons, possibly as a result of Ca^2+^-mediated mitochondrial toxicity (Du et al., 2022). Previous work (Tovar-y-Romo et al., 2012) showed that L-type VGCC activity was necessary for spine loss elicited by EFV and its 8-OH metabolite. To determine if L-type VGCC activity was necessary for the synapse loss described here we co-applied the L-type VGCC inhibitor nimodipine (Nim, 10 µM) with each ARV that produced synapse loss. Of the four ARVs assessed, only BIC-induced synapse loss was reversed significantly by L-type VGCC blockade. The 22% ± 3% decrease in PSDs elicited by 10 µM BIC was attenuated to a 10% ± 3% loss in the presence of 10 µM Nim (*p* < 0.05) (Figures 6A, E–G). The representative images in Figures 6E–G show that BIC evoked synapse loss in the absence of gross toxicity and that treatment with Nim attenuated this loss. Nim produced a slight attenuation of the loss of PSDs evoked by ETR, LEN, and NFV that was not statistically different from that evoked by the ARVs alone (Figures 6B–D). The effects of Nim, particularly in the case of ETR may result from an increase in synapses produced by Nim alone.

### The role of glutamatergic signaling in ARV-induced synapse loss

**FIGURE 7:**
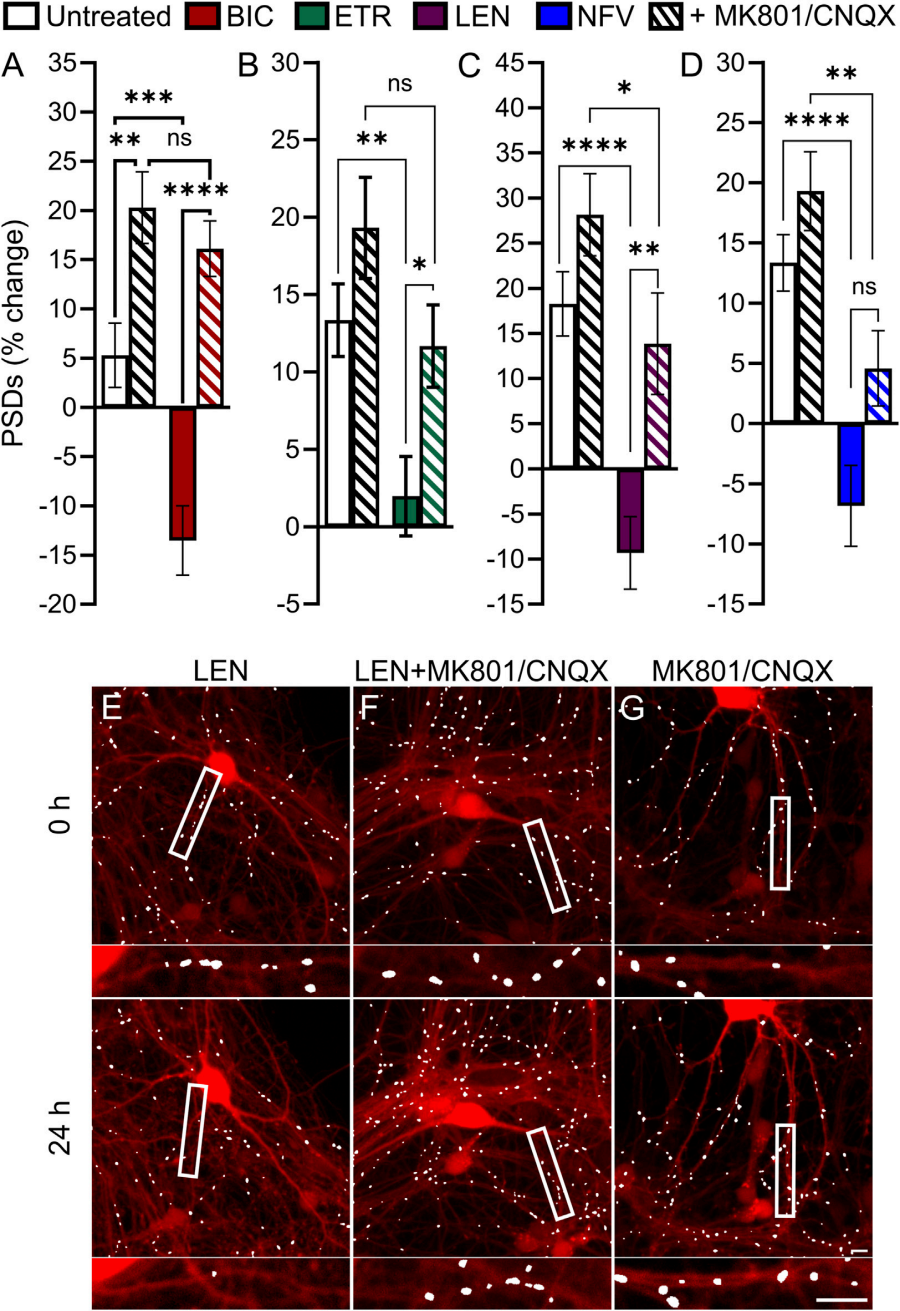
BIC, ETR, and LEN induce synapse loss through glutamatergic signaling. **(A–D)** Bar graphs show change in PSDs following 24 h exposure to the treatments indicated. MK801 and CNQX were administered in combination, both at a concentration of 10 µM. All data are presented as mean ± SEM. *p < 0.05, **p < 0.01, ***p < 0.001, ****p < 0.0001. Each treatment group includes images collected from 12 to 15 wells from 3 separate platings of primary neuronal cultures. **(A)** BIC induced synapse loss was prevented by treatment with MK801/CNQX. Two-way ANOVA with Sídák’s multiple comparisons test; n = 70–96 BIC treatment F_(1,319)_ = 11.50, *p* < 0.001, MK801/CNQX treatment F_(1,319)_ = 43.39, *p* < 0.0001, interaction F_(1,319)_ = 4.668, *p* < 0.05. **(B)** ETR induced synapse loss was protected by MK801/CNQX. Welch’s ANOVA with Games-Howell’s multiple comparisons test, n = 75–82, F_(3,172.2)_ = 6.569, *p* < 0.001. **(C)** LEN induced synapse loss was partially prevented by MK801/CNQX. Kruskal–Wallis test with Dunn’s multiple comparisons n = 61–67, Χ^2^_(4)_ = 37.70, *p* < 0.0001. **(D)** NFV induced synapse loss was not prevented by MK801/CNQX. Welch’s ANOVA with Games-Howell’s multiple comparisons test, n = 75–83, F_(3,171.6)_ = 12.50, *p* < 0.0001. Representative processed images show ROIs before (t = 0) and after 24 h treatment with LEN **(E)**, LEN + MK801/CNQX **(F)**, and MK801/CNQX **(G).** Insets display enlarged images of the boxed regions. Scale bars indicate 10 µm.

Based on previous work indicating that PIs may disrupt excitatory amino acid transporter 2 function leading to an increase in extracellular glutamate (Vivithanaporn et al., 2016), we investigated the contribution of activated glutamate receptors to ARV-induced synapse loss. Application of 10 μM MK801, an NMDA receptor antagonist, and 10 µM CNQX, an AMPA receptor antagonist, was used to block ionotropic glutamate receptors (Figure 7). Interestingly, of the 4 ARVs tested only the synapse loss induced by the PI NFV was not significantly attenuated by MK801/CNQX (Figure 7D). The loss of PSDs evoked by BIC, ETR and LEN were all attenuated significantly by blockade of glutamate receptors (Figures 7A–C). BIC induced a 13% ± 3% decrease in PSDs that was reversed to a 16% ± 3% gain of PSDs in the presence of MK801/CNQX (Figure 7A). ETR stabilized the number of PSDs over 24 h in this assay (relative to a 13% ± 2% gain in PSDs in untreated ROIs) creating a significant decrease relative to untreated. The number of PSDs increased by 11% ± 2% when ETR was applied in the presence of MK801/CNQX (Figure 7B), a significant increase relative to ETR alone. Furthermore, in the case of BIC and ETR, the synapse protection afforded by MK801/CNQX was essentially complete. The 9% ± 4% loss of PSDs produced by 10 µM LEN was reversed to a 13% ± 5% gain of PSDs in the presence of MK801/CNQX. While the MK801/CNQX did not completely reverse the synapse loss evoked by LEN, there was a significant difference from LEN alone.

### cART regimens do not synergistically induce synapse loss

**FIGURE 8:**
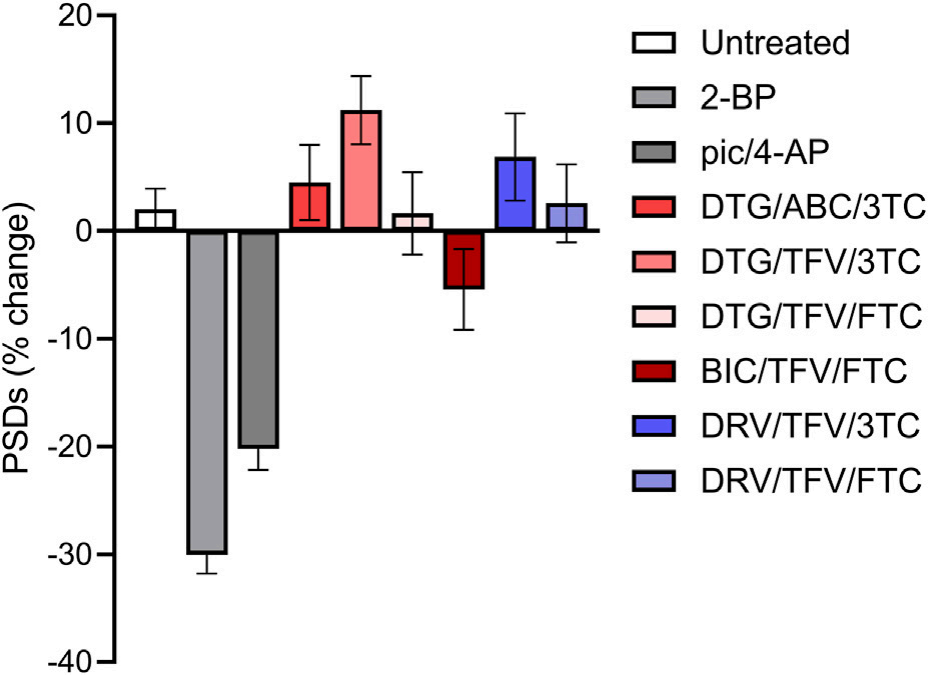
Clinically relevant cART regimens did not synergize to induce excitatory synapse loss. Bar graphs show changes in PSDs following 24 h exposure to treatments indicated. All drugs were administered at a concentration of 10 µM. All data are presented as mean ± SEM. Each treatment group includes images collected from 12 to 15 wells from 3 separate platings of primary neuronal cultures. n = 62–208. cART treatments were compared to their matched controls using a Mann-Whitney, *t*-Test, or Welch’s *t*-Test as appropriate. No tests reached statistical significance.

We next determined if combining ARVs would increase the amount of synapse loss observed. There are many U.S. Food and Drug Administration approved cART regimens. We focused on the 6 cART regimens recommended by the NIH for initial therapy (Office-of-AIDS-Research-Advisory-Council, 2022). BIC/TFV/FTC, DTG/ABC/3TC, DTG/TFV/FTC, DTG/TFV/3TC, DRV/TFV/FTC, and DRV/TFV/3TC, were applied at a 10 µM final concentration for each drug. As shown in Figure 8, no cART regimen induced changes in PSDs relative to the actions of the drugs individually (Figure 3). In contrast to the synapse loss induced by treatment with BIC alone, the BIC/TFV/FTC combination produced only 5.4% ± 3.8% loss which was not significantly different from untreated (Mann-Whitney U = 3373, *p* = 0.11).

### 8-OH-EFV induces excitatory synapse loss

**FIGURE 9:**
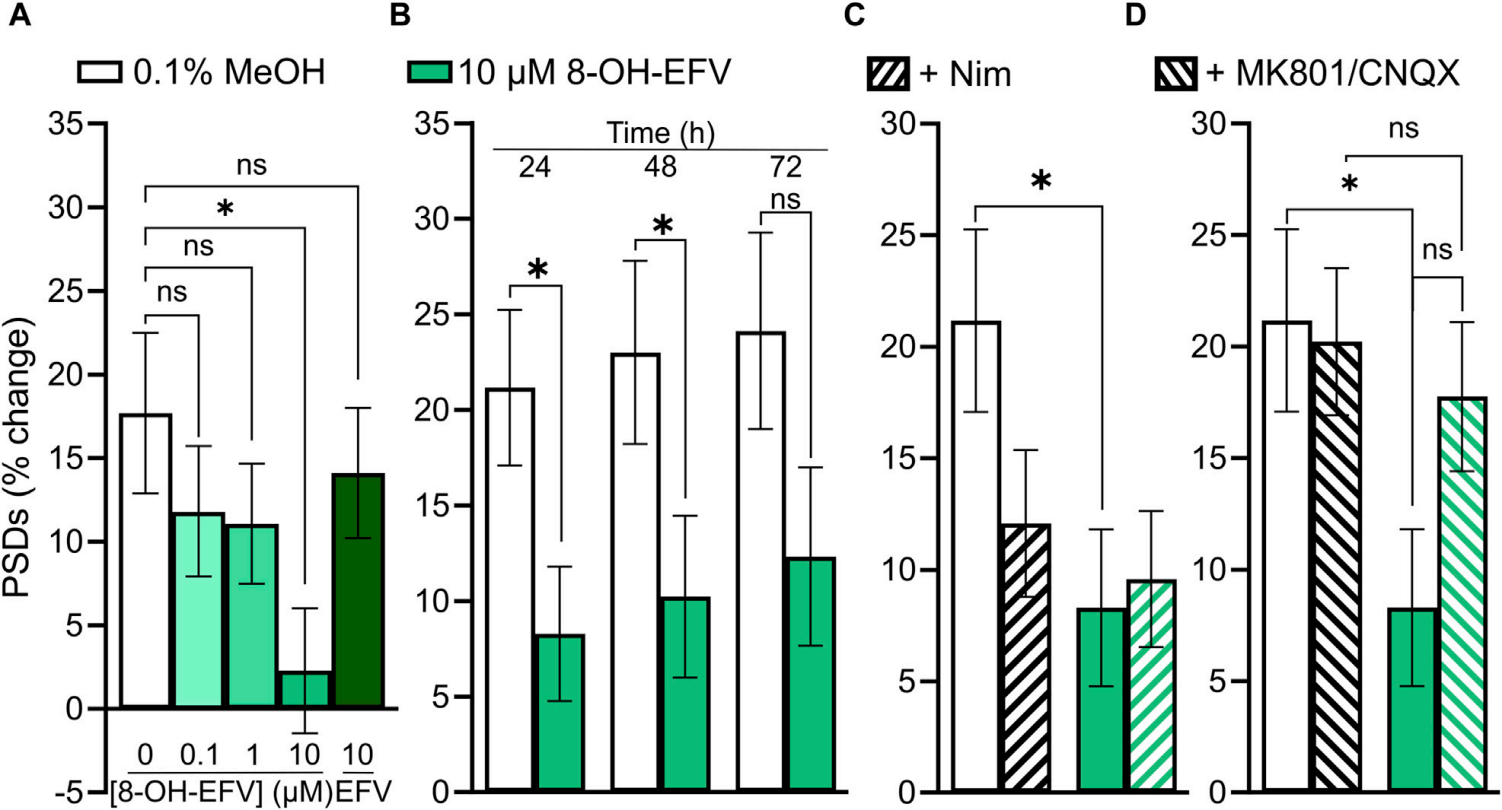
10 µM 8-OH-EFV induces excitatory synapse loss. **(A–D)** Bar graphs show changes in PSDs following 24 h exposure to treatments indicated. All data are presented as mean ± SEM. *p < 0.05. Each treatment group includes images collected from 12 to 15 wells from 3 separate platings of primary neuronal cultures. **(A)** 10 µM 8-OH-EFV, but not 10 µM EFV, induced a significant decrease in PSDs following 24 h exposure to drug. n = 52–80. Kruskal–Wallis test with Dunn’s post-tests, Χ^2^_(5)_ = 9.269, *p* = 0.055. **(B)** 8-OH-EFV induced decreases in PSDs persist to 48 h. 24 h: n_unt_ = 66, n_8-OH_ = 80, Mann-Whitney test U = 2011, *p* < 0.05. 48 h: n_unt_ = 63, n_8-OH_ = 78, Mann-Whitney test U = 1978, *p* < .05. 72 h: n_unt_ = 62, n_8-OH_ = 77, *t*-Test t_(137)_ = 1.698, *p* = 0.092. **(C)** Nimodipine does not protect against 8-OH-EFV induced decreases in PSDs. n = 66–84. Kruskal–Wallis test with Dunn’s post-tests, Χ^2^_(4)_ = 8.031, *p* < 0.05. **(D)** MK801/CNQX prevents 8-OH-EFV induced decreases in PSDs. n = 66–86. Kruskal–Wallis test with Dunn’s post-tests. Χ^2^_(4)_ = 7.769, *p* = 0.051.

As discussed earlier, we anticipated that EFV would decrease the number of PSDs based on previous work. When we failed to see an effect of EFV, we investigated the EFV metabolite 8-OH-EFV which has been shown to be tenfold more potent in evoking synaptic spine loss than its parent drug (Tovar-y-Romo et al., 2012). In previous studies this compound was delivered in 0.1% methanol. In our assay, we found that 0.1% methanol increased the number of synapses relative to untreated cultures. Thus, we used a 0.1% methanol vehicle control for comparison purposes. A statistically significant prevention-of-gain in PSDs was induced by 10 µM 8-OH-EFV (4% ± 2% gain v. 13% ± 3% gain in 0.1% MeOH) while 10 µM EFV had no effect (11% ± 3% gain in PSDs) (Figure 9A). The net decrease in PSDs produced by 8-OH-EFV persisted for 72 h although the decrease was no longer statistically significant at the 72 h time point (*p* = 0.092) (Figure 9B). In contrast to previously reported work, we did not see robust protection from 8-OH-EFV induced decreases in PSD number when the ARV metabolite was administered in the presence of the L-type VGCC inhibitor Nim (Figure 9C). While 8-OH EFV in the presence of Nim did not induce a significant decrease in PSDs relative to the vehicle control, there was a strong trend towards loss (*p* = 0.06). However, interpretation of this experiment is complicated by the decrease in PSDs produced by Nim alone. As shown in Figure 9D, MK801/CNQX prevented the synapse loss induced by 8-OH EFV. The change in PSDs evoked by 8-OH EFV in the presence of MK801/CNQX was not different from cultures treated with vehicle or MK801/CNQX alone. However, the apparent protection was not different statistically from the PSD loss produced by the 8-OH EFV alone, suggesting that glutamate signaling blockade may produce incomplete protection (Figure 9D).

## Discussion

The persistence of HAND despite virological suppression in PLWH suggests that some other factor, such as ARVs themselves, may contribute to neurocognitive impairments (Ellis, 2010; Underwood et al., 2015). The loss of synaptic connections correlates with cognitive impairment in HAND (Ellis et al., 2007). Here we have shown that ARVs from 4 classes induce excitatory synapse loss, consistent with the idea that ARV-induced synapse loss contributes to the prevalence of HAND. We screened 25 ARV drugs to determine which compounds induced excitatory synapse loss in a high content, automated synapse imaging assay. Five ARVs were identified as inducing excitatory synapse loss: the ISTI BIC, the NNRTI ETR, the CI LEN, and the PIs NFV and SQV. This report is the first to describe synapse loss evoked by BIC, NFV, SQV, and LEN.

ARVs are associated with adverse nervous system events, including peripheral neuropathy and neuropsychiatric effects (Abers et al., 2014). These neuropsychiatric effects range from unusually vivid dreams to insomnia, depression, and suicidality. As some neuropsychiatric illnesses are associated with synapse loss, this further bolsters our belief that ARVs may exert some of these effects through synapse loss (Bennett, 2009; Duman, 2014b; a;Holmes et al., 2019). The NNRTI EFV is particularly known for producing adverse neuropsychiatric events, which affect up to half of all patients and are a frequent reason for discontinuation of EFV-containing regimens (Arendt et al., 2007; Leutscher et al., 2013). Furthermore, neuropsychiatric symptoms have been reported for all ISTIs (Kolakowska et al., 2019) and DTG has been associated with the onset of novel depression (Hoffmann and Llibre, 2019). However, in our assay DTG did not evoke synaptic changes. Discontinuation rates due to neuropsychiatric symptoms are generally elevated in DTG-containing regimens relative to those containing BIC (Pérez-Valero et al., 2023) which produced robust synapse loss in our assay. Thus, the automated synapse loss assay does not capture all mechanisms that lead to adverse neuropsychiatric symptoms and the induction of synapse loss was not uniform across drugs within a given class. Some studies have suggested that increased CNS penetrance by ARVs may worsen cognitive function (Marra et al., 2009; Caniglia et al., 2014). Additionally, multiple studies have shown that ARV discontinuation improves cognitive function in some patients (Robertson et al., 2010; Underwood et al., 2015). This remains controversial, however, as other studies have indicated that patients taking cART regimens with greater CNS penetrance see no change—or even improvement in—cognitive function (Smurzynski et al., 2011; Eisfeld et al., 2013; Carvalhal et al., 2016). The most parsimonious explanation for these discordant observations is that HAND can result from a variety of factors, some induced by HIV in the CNS and others produced by ARVs.

After establishing which ARVs induced synapse loss at the 10 µM concentration used in the initial screen (Figure 3), we assessed the concentration-dependence of synapse loss for those drugs identified as hits. All 4 compounds elicited excitatory synapse loss in the range of their clinically measured total plasma concentrations (C_max_, Table 1). However, all 4 drugs are highly bound to plasma proteins and thus only LEN induced excitatory synapse loss at the clinically relevant concentrations we estimate to be free in plasma (Table 1), highlighting a potential neurotoxicity risk posed by this novel capsid inhibitor (Link et al., 2020; U.S.FDA, 2022). At the time of writing, no data on LEN concentrations in the CSF have been published, leaving open the question on whether this drug may be reaching synaptotoxic concentrations in patients’ nervous systems. The LEN concentration response relationship was unusual in that the 10 nM drug concentration evoked more synapse loss than either the 1 or 100 nM concentration. This peak effect at 10 nM was seen in all 3 replicate experiments each with fresh drug made up from serial dilutions in vehicle. We examined the stocks carefully for precipitate because this drug is administered subcutaneously as a depot that precipitates and is slowly released over months (Subramanian et al., 2023). Thus, while reproducible, we cannot explain the unusual LEN concentration response curve in Figure 4C. LEN clearly evokes synapse loss at concentrations comparable to free plasma drug concentrations that might be expected during initial oral dosing prior to administration of a subcutaneous depot (U.S.FDA, 2022).

BIC, ETV and NFV produced synapse loss only at concentrations comparable to C_max_; it is possible that synapse loss is not the cause underlying the adverse CNS effects of these drugs or other factors may be relevant. Drug displacement of highly protein bound ARVs by administration of other drugs (whether medical or recreational) risks increasing plasma concentrations to potentially toxic levels. Additionally, as HIV infection can damage the integrity of the blood-brain barrier (McRae, 2016), CSF concentrations of ARVs risk rising to potentially toxic levels. This may be particularly relevant for LEN due to its ability to induce synapse loss at clinically relevant concentrations free in plasma (Table 1). Patients with underlying neuropsychiatric disorders may be more sensitive to synapse loss induced by ARVs. Psychiatric illnesses have been associated with synapse loss (Bennett, 2009; Duman, 2014b; a;Holmes et al., 2019); thus, our work suggests that ARV-induced synapse loss may synergize with disease-associated synaptic deficits to contribute to ARV-associated neuropsychiatric effects. Overall, the synapse loss evoked by BIC, ETV and NFV should be viewed as a potential contributor to adverse CNS effects of these drugs under special circumstances that might result from elevated plasma drug levels or enhanced CNS sensitivity.

ARVs reduce mother-to-child-transmission of HIV (Schnoll et al., 2021) and drugs that produce synapse loss might have neurodevelopmental consequences (Foster et al., 2023). Umbilical cord-to-maternal plasma concentration ratios for BIC, ETV, and NFV are 1.46, 0.52, and 0.19, respectively (Mulligan et al., 2016; Eke et al., 2019; Bukkems et al., 2021). While none of the ARV drugs that produced synapse loss in the automated synaptic imaging assay are recommended during pregnancy (Schnoll et al., 2021), the assay provides an *in vitro* method to evaluate drugs being considered for such use.

In time course experiments BIC, ETR, LEN, and NFV caused synapse loss that persisted for 3 days. None of the drugs displayed a significant increase in synapse loss over time suggesting that their effects on synaptic networks reach steady state within 24 h. This observation is consistent with the role of ionotropic glutamate receptors in the mechanism of synapse loss for BIC, LEN, and ETR because neural networks compensate for excess excitatory synaptic activity by downscaling synaptic number and/or strength (Turrigiano, 2012). This suggests that the observed synapse loss may actually be a coping mechanism to protect the network from excitotoxicity. While a 3 days exposure does not approximate the lifelong ARV treatment PLWH receive, our data raise the possibility that prolonged drug exposure would not necessarily produce cumulative synaptic loss over time. Taken together, the time course and concentration-response data suggest that chronic use of ARVs could produce a persistent decrease in the number of excitatory synapses if CSF levels of the ARV were significantly elevated.

We identified two potential mechanisms underpinning ARV-induced synaptotoxicity. VGCC activation was shown previously to contribute to EFV-induced hippocampal spine loss (Tovar-y-Romo et al., 2012), and we believed that this mechanism might expand beyond just that of the NNRTIs. While we anticipated that VGCC inhibition might protect against synapse loss induced by the NNRTI ETR, the inhibitor of L-type VGCC Nim did not reverse the decrease in synapses produced by ETR. We found that co-application of Nim protected only against BIC-induced synapse loss, and this protection was incomplete. The finding that BIC-induced synapse loss has a VGCC component is interesting as the cART regimen Triumeq (DTG/ABC/3 TC) has been found to increase prefrontal pyramidal neuron excitability through low voltage activated L-type VGCCs, suggesting that as a class ISTIs might activate Ca^2+^ channels (Chen et al., 2020). Additionally, we assessed whether excessive glutamatergic signaling contributed to ARV-induced synapse loss as PIs have been shown to disrupt astrocytic excitatory amino acid transporter 2 expression and would thus elevate extracellular glutamate concentrations (Vivithanaporn et al., 2016). Interestingly, we found that the synapse loss produced by BIC, ETR, and LEN was attenuated by blocking ionotropic glutamate receptors with the NMDAR inhibitor MK801 and the AMPAR inhibitor CNQX. Only the synapse loss produced by NFV was unaffected by either L-type VGCC inhibition or blockade of glutamate signaling.

Other mechanisms of neurotoxicity have been associated with ARV drugs. In striatal presynaptic nerve terminals ARVs reduced energetic reserve, although they did not produce synapse loss in the automated synaptic imaging assay (Stauch et al., 2017). This may be explained by the lack of ARV effects on the bioenergetics of hippocampal cultures. However, reductions in glutamate release that occur when presynaptic ATP is depleted would be predicted to cause an increase in synaptic density as part of an adaptive upscaling in the assay used here. Indeed ABC, one of the ARVs that impaired energetic reserve in striatal nerve terminals produced a significant increase in synaptic density (Figure 3). We replicated the previous finding that 8-OH-EFV, a metabolite of EFV that lacks antiviral activity, induced synapse loss. We did not see any protection from 8-OH-EFV-induced synapse loss by treatment with Nim in our assay. Instead, we saw a trend towards protection when 8-OH-EFV was co-administered with MK801/CNQX. The robust synapse loss induced by BIC (Figures 3–7) was diminished when tested in combination with TFV and FTC. It is possible that TFV and FTC act to reduce the toxicity of BIC, although this is speculative as a potential mechanism for a protective effect of these drugs is not apparent. Overall, these data suggest that mechanisms of ARV-induced synapse loss can be ARV-specific.

Previous reports have described ARV induced neurotoxicity. In contrast to previously reported studies (Tovar-y-Romo et al., 2012), we did not see EFV-induced synapse loss or neuronal toxicity in our screen. However, the 8-OH metabolite of EFV induced synapse loss consistent with the loss of spines described by Tovar-y-Romo et al. (2012). The synapse loss induced by 8-OH-EFV in this study occurred at concentrations well above the 10 nM concentration that evoked a loss of dendritic spines or that is present in the CSF of EFV-treated patients (Tovar-y-Romo et al., 2012). We anticipated results from automated synaptic imaging to be similar to results from counting dendritic spines. However, in the assay employed here EFV produced neither synapse loss nor cell death over 24 h even though cell culture conditions used by the two studies are quite similar. It is possible that dendritic spines represent a subset of synapses that are particularly sensitive to EFV and its metabolite and thus the synapses visualized by the imaging assay which include non-spiny synapses on dendritic shafts (Benson et al., 1994; Fortin et al., 2014) might be less sensitive to EFV. Robertson et al. (2012) reported ABC-induced neuronal toxicity; we saw a statistically significant increase in synapses following a 10 µM application of the drug. The increase in synapses produced by ABC alone and in combinations may be an early response to presynaptic energy failure that develops into more extensive toxicity over time. Our observation of ETR-induced synapse loss is in line with the neural toxicity observed by the same group (Robertson et al., 2012). Work from Smith et al. has shown that prolonged, 7 days exposures to elvitegravir, DTG/TDF/FTC, EVG/TDF/FTC, and TDF/FTC can reduce synapse density relative to controls in hiPSC-derived neurons but did not observe synapse loss or neuronal toxicity within 24 h (Smith et al., 2022). The use of primary rodent neurons *versus* hiPSC-derived neurons and the shorter incubation time may account for the lack of synapse loss detected in the assay described here.

Synaptic networks in culture are highly dynamic which is advantageous for studying adaptation to drug or other treatments. For example, the automated synapse loss assay employed here can detect synapse loss evoked by adaptations to decreased inhibitory tone, increased synaptic or extrasynaptic glutamate, or subtle changes in membrane potential produced by effects on metabolism or ion channels. However, this dynamic nature also adds to the variability of the assay. A particularly noticeable form of variation was the range of synaptogenesis observed in untreated control wells. Optimal timing of the maturation of the network for each unique cell plating varied by 1-2 DIV. Thus, we compared all control treatments and drug evoked responses to matched untreated ROIs from the same plating. This approach captured drug induced synapse loss regardless of the level of synaptogenesis in that particular culture. With the exception of the ETR-evoked synapse loss in the initial screen the net loss of synapses was consistent for ETR and other treatments. We chose to present the separate untreated control and drug responses rather than the net differences, to make clear that drug effects are relative to a baseline unique to each 96-well plate. Synaptic networks are constantly changing and thus continuously undergoing synapse loss and formation. We did not detect significant changes in the net synapse loss produced by the drugs studied here based on the level of synaptogenesis in untreated control wells, although our experiments were not designed to differentiate increased synapse loss from decreased synaptogenesis. Gross toxicity also complicates the assay because cell death creates a total loss of synapses rather than a graded loss more relevant to neuropsychiatric disorders and early stage neurodegenerative disease. Gross toxicity contributed to some of the responses observed to high concentrations of NFV, but the other ARV drugs did not elicit cell death in this assay. Finally, the consistency of an assay is related to the sensitivity of the detected effects. In the case of drugs only active at Cmax it is possible that these ARVs were near the limit of detection and thus the modest effect size reduced statistical power.

The automated synapse loss assay employed here has certain limitations. Microglia and astrocyte populations are reduced in our cell culture system, preventing us from assessing indirect synaptotoxicity that may occur in the presence of glial cells. Future studies to assess ARV-induced synapse loss in cultures containing glial cells that could manifest a full neuroinflammatory response are warranted, especially as some ARVs have been found to induce inflammatory cytokine release (Wu et al., 2017). Additionally, we assessed synapse loss with only ARVs present, while PLWH would have some base level of soluble viral proteins present in the CNS at the time of therapy initiation. HIV proteins such as TAT and gp120 affect synaptic function. Testing ARVs in the presence of viral proteins might better replicate the neural environment in patients with HAND, and could unmask synergistic effects (Dawson and Dawson, 1994; Kaul and Lipton, 1999; Perez et al., 2001; Nath, 2002; Kim et al., 2008; Shin and Thayer, 2013; Thangaraj et al., 2018). The studies described here were performed on primary rodent neurons. Clearly human neurons might express unique drug sensitivities lacking in synaptic networks derived from rodent neurons. Studies using hiPSC-derived neuron cultures, which have largely intact signaling networks (Autar et al., 2022; Smith et al., 2022; Asher et al., 2023), could also increase translatability of such research.

The ever-evolving nature of HIV results in ARV drug resistance that ultimately results in virological failure (McCluskey et al., 2019), highlighting the importance of constant development of new drugs. The automated synaptic imaging assay used here may prove valuable in screening drug candidates for potential synaptic toxicity as part of preclinical development.

## Data Availability

The raw data supporting the conclusion of this article will be made available by the authors, without undue reservation.
